# Elafin and its precursor trappin‐2: What is their therapeutic potential for intestinal diseases?

**DOI:** 10.1111/bph.15985

**Published:** 2022-11-28

**Authors:** Céline Deraison, Chrystelle Bonnart, Philippe Langella, Karine Roget, Nathalie Vergnolle

**Affiliations:** ^1^ IRSD, Université de Toulouse, INSERM, INRAE, ENVT, Univ Toulouse III ‐ Paul Sabatier (UPS) Toulouse France; ^2^ Université Paris‐Saclay, AgroParisTech, Micalis Institute, INRAE Jouy‐en‐Josas France; ^3^ Nexbiome Therapeutics Clermont‐Ferrand France; ^4^ Department of Physiology and Pharmacology University of Calgary Calgary Alberta Canada

**Keywords:** colitis, elafin, inflammation, inflammatory bowel disease, protease, trappin

## Abstract

Elafin and its precursor trappin‐2 are known for their contribution to the physiological mucosal shield against luminal microbes. Such a contribution seems to be particularly relevant in the gut, where the exposure of host tissues to heavy loads of microbes is constant and contributes to mucosa‐associated pathologies. The expression of trappin‐2/elafin has been shown to be differentially regulated in diseases associated with gut inflammation. Accumulating evidence has demonstrated the protective effects of trappin‐2/elafin in gut intestinal disorders associated with acute or chronic inflammation, or with gluten sensitization disorders. The protective effects of trappin‐2/elafin in the gut are discussed in terms of their pleiotropic modes of action: acting as protease inhibitors, transglutaminase substrates, antimicrobial peptides or as a regulator of pro‐inflammatory transcription factors. Further, the question of the therapeutic potential of trappin‐2/elafin delivery at the intestinal mucosa surface is raised. Whether trappin‐2/elafin mucosal delivery should be considered to ensure intestinal tissue repair is also discussed.

**Abbreviations**
AP‐1: activator protein‐1
F2: thrombin or factor‐2
HIV: immunodeficiency virus
IBD: inflammatory bowel diseases
SLPI: secretory leukocyte protease inhibitor
SNPs: single nucleotide polymorphisms
WAP: whey acidic protein

## 1 INTRODUCTION

The intestinal mucosa is constantly exposed to, and co‐exists with a wide range of antigens, commensals or infectious pathogens, while assuming complex biological functions that include digestion, nutrient intake, or specialized exchanges. To accommodate these functions, the intestinal mucosa has evolved unique and sophisticated defence mechanisms that ensure tissue homeostasis. These mechanisms are capable of tolerating beneficial or neutral entities, while reacting against harmful, damaging microbes or products, to eventually favour a return to the tissue equilibrium and physiological functions associated with a healthy state.

Such equilibrium is disrupted in intestinal pathologies and most particularly in pathologies that are associated with chronic inflammation. In diseases such as Crohn's disease or ulcerative colitis, two chronic conditions, classed under the common term of inflammatory bowel diseases (IBD), as well as in celiac disease, an immune response against food and microbial antigens together contribute to tissue destruction. Although they have different aetiologies, these diseases have, in common, a disrupted microbiota ecology, invading pathobionts, an overactive immune system, and a defective intestinal barrier function. In such pathologies, the mechanisms that normally control the mucosal inflammatory response and the return to homeostasis are overwhelmed, causing considerable damage to intestinal mucosa tissues. Among the endogenous mechanisms that have been described to control mucosal immunity and to prevent excessive tissue damage, different classes of proteins have been described. One in particular, elafin, and its precursor form, trappin‐2, appear as very peculiar and pleiotropic mediators, able to control, simultaneously, excessive tissue proteolysis, inflammatory cell infiltration and activation, and microbial invasion.

In this review, we discuss the evidence that point to a role for trappin‐2/elafin in intestinal pathologies, with particular emphasis on inflammatory diseases. The review aims to provide a systematic understanding and a critical analysis of the pleiotropic effects that could be exerted by trappin‐2/elafin in gut pathologies.

## 2 STRUCTURE, MODE OF ACTION, AND CONTROL OF TRAPPIN‐2/ELAFIN

### 2.1 Trappin‐2/elafin structure: A three‐in‐one product

**FIGURE 1:**
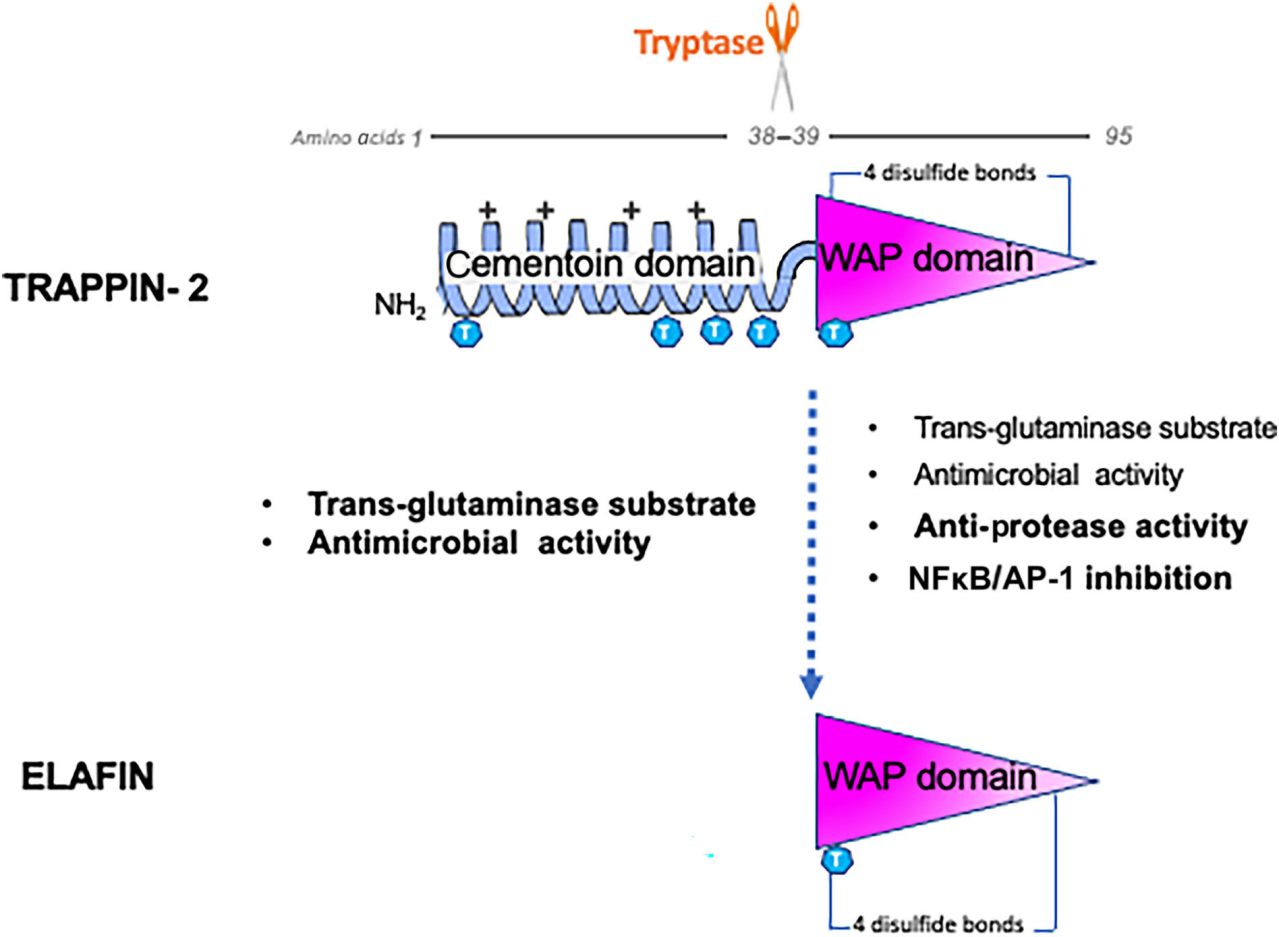
Diagram of trappin‐2 and elafin with location of their biological functions. The elafin domain (WAP domain) is released from trappin‐2 (containing both cementoin and WAP domains) after proteolytic cleavage at amino acid 38. The cementoin domain has a α‐helix structure (blue ribbon), and the WAP domain contains the anti‐protease activity (pink triangle). Associated biological functions are shown on the left for the cementoin domain and on the right for the WAP domain. Blue hexagons with T sign correspond to the repeated motifs rich in Gln and Lys residues, substrate of transglutaminase. Amino acids numbers are in grey and italics.

Elafin and its precursor trappin‐2 are inhibitors of human serine proteases and members of the chelonianin family (from the MEROPS classification database), which also includes the secretory leukocyte protease inhibitor (SLPI). Both members of this family have peptide sequences that have similarities with the sequence of an inhibitor isolated from the chelonian sea turtle, thereby explaining this family name. Elafin (6‐kDa) was first purified in the 1990s from the skin of patients with psoriasis and was characterized as a protein with elastase inhibitor properties (Schalkwijk et al., 1990; Wiedow et al., 1990). It was the cloning of elafin cDNA, which revealed that the molecule was longer than expected revealing a precursor form that contains an additional N‐terminal domain, distinct from the domain with the protease inhibitor function. The whole protein (the precursor) was then named trappin‐2 (12‐kDa). This name stands for TRansglutaminase substrate and whey Acidic ProteIN domain and it reflects the two different domains of trappin‐2 (see Figure 1) which are

‐ a flexible N‐terminal domain of 38 amino acids, also called the cementoin domain, which is a substrate for transglutaminase. This domain contains several repeated motifs rich in Gln and Lys residues (four GQDX‐VK consensus sequences that are transglutaminase substrates), which after the action of transglutaminase, serve to anchor the whole molecule to extracellular matrix proteins (Nara et al., 1994). The cementoin domain also bears a high net positive charge that is believed to play a role in antimicrobial properties of the molecule (Baranger et al., 2008).
‐ A C‐terminal domain, which contains a whey acidic protein (WAP)‐type domain, and which is endowed with the anti‐protease activity. This domain by itself corresponds to the protein elafin. This WAP domain also contains one transglutaminase substrate motif.

Many different proteases could catalyse the proteolytic cleavage of trappin‐2 into elafin, although serine and cysteine proteases are more effective than metalloproteinases in releasing elafin from its precursor form, trappin‐2. Mast cell tryptase has been suggested to play the role of physiological elafin‐releasing enzyme (Guyot, Zani, Berger, et al., 2005). In an inflammatory context, where tissue proteolytic activity is markedly increased (Vergnolle, 2000, 2016), many different candidate proteases could be responsible for trappin‐2 cleavage and the release of elafin. However, it is still not yet clear which form of the protein (trappin‐2, elafin, or both) is associated with any specific physiological or pathophysiological conditions.

**FIGURE 2:**
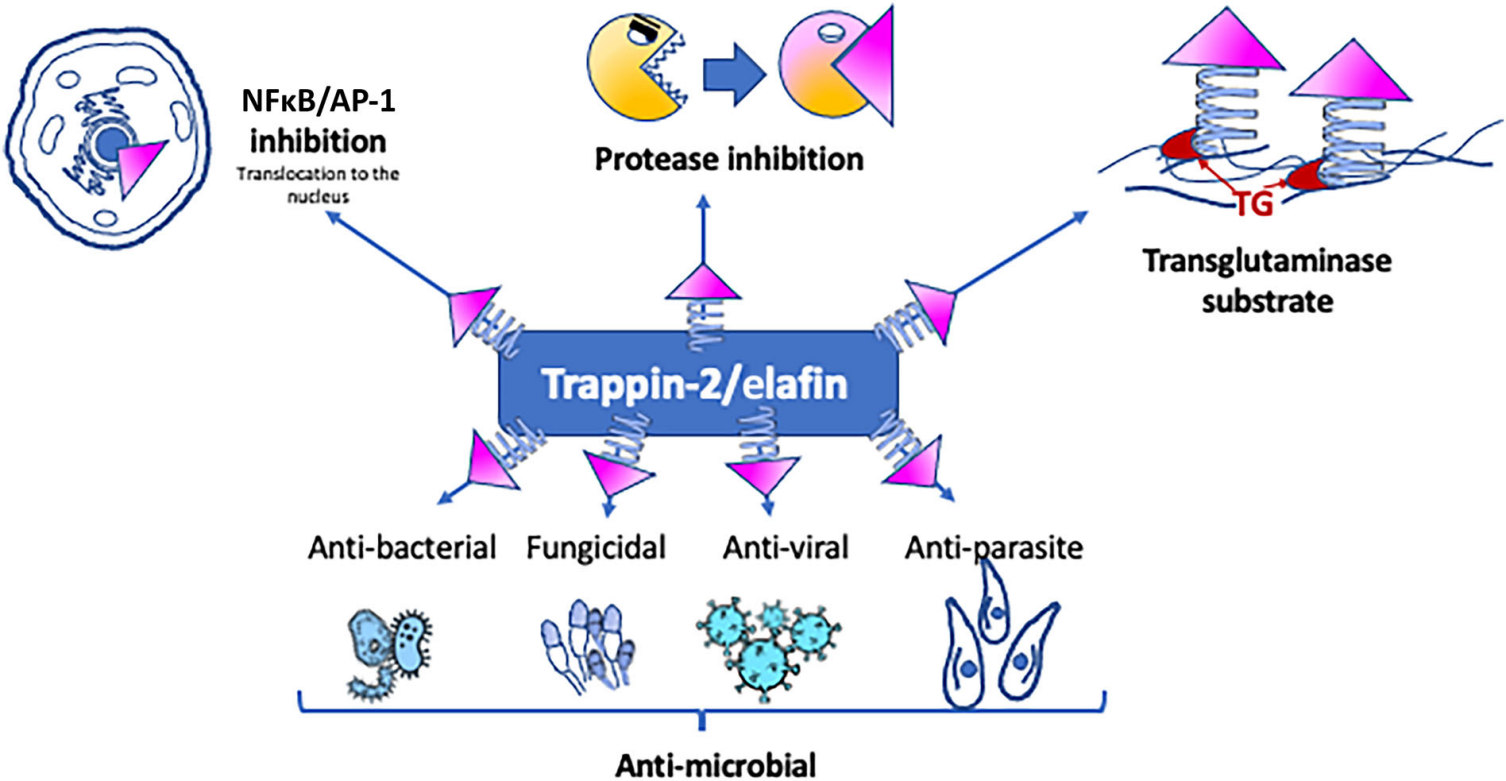
Pleiotropic mechanisms of action of trappin‐2/elafin. The anti‐protease WAP domain (pink triangle) is important to control proteolytic activity and interferes with NF‐kB/AP‐1 pathways. Trappin‐2/elafin has demonstrated anti‐microbial properties against bacteria but also potentially against some fungi, viruses and parasites. Trappin‐2/elafin contains substrate motifs for transglutaminase.

Whether trappin‐2 or elafin is considered, many biological activities have been associated with one or both entities. Such biological properties can be carried by the N‐terminal cementoin domain, by the C‐terminal elafin domain, or by the combination of both, thereby proposing for trappin‐2 the concept of a three‐in‐one molecule. All the biological properties that have been described for trappin‐2/elafin are presented here below and are summarized in Figure 2.

### 2.2 Protease inhibition

Elafin binds to the catalytic site of the target protease, thereby blocking the access of substrates in a non‐covalent manner. This mechanism of action differs from that of the serpins, which covalently bind target proteases leading to a permanent inactivation. The protease inhibitor region is located between amino acids Leu20(P5) and Leu26(P2′). The protease inhibitory loop is connected to the central β‐sheet by two external peptide chains and is stabilized by the fourth disulfide bond. The scissile peptide bond is between Ala24(P1) and Met25(P1′). Hydrogen bonding and van der Waals interactions stabilize the interaction between protease and inhibitor. The crystal structure of elafin in complex with porcine pancreatic elastase has been resolved showing that the Ala24 carbonyl group projects into the oxyanion hole created by nitrogen atoms of Gly193 and Ser195 from the catalytic triad of elastase, thus inhibiting catalysis (Tsunemi et al., 1996).

The major characteristic of elafin and its precursor trappin‐2 as protease inhibitors is their very restricted spectrum of antiprotease activity. In contrast to most other protease inhibitors, trappin‐2/elafin only inhibits relatively few endogenous proteases. Considering the proteases present in the gastrointestinal tract, elafin could inhibit the chymotrypsin‐like elastase family member 2A (also called Ela2A from the CELA2A gene), an epithelial form of elastase (Motta, Rolland, et al., 2021). In inflammatory conditions, elafin could inhibit, in the gut, the neutrophil elastase (*ELANE* gene) and the neutrophil proteinase 3 (also called PR3, from the *1PRTN3* gene) (Zani et al., 2004). Kallikrein‐7 (*KLK7* gene), a chymotrypsin‐like protease overexpressed in human colonic tumours, can also be inhibited by elafin (Franzke et al., 1996; Oikonomopoulou et al., 2006) (see Table 1). Both the full‐length precursor trappin‐2 and the cleaved form, elafin, retain such anti‐protease activity for the same proteases and within the same molecular concentration range (Moreau et al., 2008). This indicates that the cementoin domain of trappin‐2 does not interfere with the protease catalytic pocket or the substrate binding site located on the elafin part of the molecule. Interestingly, trappin‐2/elafin affinities are very restricted to certain proteases. For instance, trappin‐2/elafin does not inhibit members of the trypsin family, coagulation factors such as thrombin (F2), or Factor 10 (F10) and it does not inhibit either mast cell tryptase or chymase (Moreau et al., 2008). Interestingly, elafin can also inhibit microbial proteases, such as the arginyl peptidase from *Pseudomonas aeruginosa* (Bellemare et al., 2008).

**TABLE 1:** Proteases present in the gut and inhibited by trappin‐2 and/or elafin

Proteases (protein name)		ELA2A	Neutrophil elastase	Proteinase‐3	Kallikrein‐7	Protease IV
Proteases (gene name)		*CELA2A*	*ELANE*	*PRTN3*	*KLK7*	Arginyl peptidase
Cellular distribution in the gut		Epithelial cells in colon	Neutrophil monocyte	Neutrophil monocyte	Epithelial cells in oesophagus	Microbiota *P. aeruginosa*
Inhibition by trappin‐2 (range of Ki)		ND	0.1 nM	0.1 nM	ND	ND
Inhibition by elafin (range of Ki)		1 nM	0.1 nM	0.1 nM	0.1 nM	ND
Up‐regulated activity in pathologies	*IBD*	+++	+++	+++	ND	+++
	*Celiac disease*	ND	+++	+++	ND	+++
	*Colon cancer*	ND	+++	+++	+++	ND

*Note*: Affinity for target proteases is indicated as inhibitory constant (K_i_). ND, not defined. Please note, the inhibition of arginyl peptidase by trappin‐2/elafin was assayed in culture media from WT and mutated *P. aeruginosa* strains. Up‐regulated levels of specific proteolytic activity in inflammatory bowel diseases (IBD), celiac disease, or colorectal cancer is indicated by “+” signs.

### 2.3 Antimicrobial activity

Elafin and its precursor trappin‐2 are considered as members of the defence molecules family. Indeed, they have demonstrated antibacterial, antifungal and antiviral activities.

The antibacterial activities of elafin and trappin‐2 have been the most studied. In vitro, antibacterial activity was demonstrated against a large array of bacteria including, but not limited to, *Staphylococcus aureus*, *Pseudomonas aeruginosa*, *Escherichia coli*, *Klebsiella pneumoniae*, and *Mycobacterium tuberculosis*. Such antibacterial activities have been demonstrated both for elafin and trappin‐2 and against both Gram‐negative and Gram‐positive bacteria. Such non‐specific nature of antibacterial activity suggests for elafin and trappin‐2 an important role in the mucosal defence arsenal. By homology to what is known about the mechanism of action of other antimicrobial peptides, the hypothetical mechanism of antimicrobial activity of trappin‐2/elafin is related to its cationic nature, as cationic charges are able to disrupt bacterial cell membranes. Indeed, trappin‐2 has a net positive charge of 7, which could interact with anionic phospholipids from bacterial membranes. In favour of such hypothesis is the fact that the antibacterial activity of trappin‐2/elafin against *P. aeruginosa* was independent of the anti‐protease activity of the elafin moiety in vitro (Simpson et al., 1999). Interestingly however, both the cementoin domain (N‐terminal) and the elafin domain (C‐terminal) can significantly kill bacteria (*P. aeruginosa* and *S. aureus* killing was tested), but the full length trappin‐2 appeared to be the most effective. The anti‐bacterial effect of the full length trappin‐2 was greater than the addition of the individual effects of the N‐terminal and the C‐terminal domains (Bellemare et al., 2010). This suggests that a critical interaction between the two domains exists to amplify the antimicrobial effects, which might not be explained by a simple cationic charge effect. Further studies are needed to decipher such mechanism.

Both trappin‐2 and elafin were identified as biomarkers of resistance in human immunodeficiency virus (HIV) infection in cervico‐lavage of seronegative, but highly exposed, sex workers (Drannik, Nag, Yao, Henrick, Jain, et al., 2012). Further studies have demonstrated that elafin was more potent than trappin‐2 at inhibiting HIV infection. This effect of elafin was apparently due for a large part, to modulation of innate immunity (Drannik, Nag, Yao, Henrick, Ball, et al., 2012; Drannik, Nag, Yao, Henrick, Jain, et al., 2012; Drannik, Nag, Yao, Henrick, Sallenave, & Rosenthal, 2012), but possible direct antiviral properties were also suggested by the work from Pfaender et al. against hepatitis C virus (Pfaender et al., 2013).

Antifungal properties have been demonstrated for trappin‐2, but not for elafin. The antifungal effects of trappin‐2 were demonstrated against *Candida albicans* and against *Aspergillus fumigatus*. A study by Baranger et al. further identified that the antifungal properties of trappin‐2 were independent from its intrinsic anti‐protease activity and were sensitive to NaCl and heparin, suggesting that the mechanism of action of trappin‐2 might depend on its cationic nature (Baranger et al., 2008).

More recently, anti‐parasitic properties have also been demonstrated for trappin‐2 against *Plasmodium berghei*, the parasite responsible for malaria (Roussilhon et al., 2017).

### 2.4 Control of innate and adaptative immunity

Elafin and trappin‐2 can inhibit inflammation and innate immunity, through the inhibition of transcription factors such as AP‐1 and NFκB, particularly in innate immune cells such as monocytes and macrophages. This mechanism involves the inhibition of the ubiquitin‐proteasome (Butler et al., 2006; Henriksen et al., 2004; Sallenave et al., 2003), but the underlying mechanisms remain unclear so far.

More recent studies have shown that elafin can also foster the resolution of inflammation. In LPS‐challenged mice, elafin accelerated resolution, associating increased apoptosis in neutrophils, and here again modulating NFκB. However, this pro‐resolving effect of elafin could be due to inhibition of neutrophil elastase, and might depend on the presence of intact (non‐degraded) annexin‐1 (Vago et al., 2016).

Elafin may protect against human neutrophil elastase‐mediated cleavage of receptors such as CD40, CD80, and CD86 on dendritic cells, which are essential for the recognition and engulfment of apoptotic cells, favouring the resolution of inflammation (Roghanian, Drost, et al., 2006). Other work has demonstrated the secretion of trappin‐2/elafin by γδ T‐cells, which could contribute to the opsonization of pathogens at mucosal surfaces (Wilkinson et al., 2011). In the context of adenovirus infection, elafin was shown to increase antibody levels and neutralization titres. Despite the fact that the mechanism is not fully understood, elafin‐treated animals exhibited a higher proliferation of splenocytes (Roghanian, Drost, et al., 2006). Altogether, these data suggest that trappin‐2/elafin secretion could modulate the numbers and activity of antigen‐presenting cells and could therefore be beneficial to protect mucosal surfaces.

### 2.5 Trappin‐2: A transglutaminase substrate

Nara et al. have demonstrated that the cementoin domain of trappin‐2 contains repeated motifs that allow transglutaminases to catalyse the formation of covalent bonds with extracellular matrix proteins (Nara et al., 1994). Further work has demonstrated that trappin‐2 can be bound covalently to laminin, fibronectin, beta‐crystallin, collagen type IV, or elastin by a transglutaminase‐dependent mechanism (Moreau et al., 2008). Interestingly, bound‐trappin‐2 fully retained its antiprotease activity and is less susceptible to degradation (Guyot, Zani, Maurel, et al., 2005). This suggests that through the action of transglutaminases, trappin‐2 could be stabilized and anchored in extracellular matrix components, where it still can exert its antiprotease activity. This covalent association of trappin‐2 to extracellular matrix suggests a possible trappin‐2‐mediated protection of matrix protein degradation.

### 2.6 Terminating trappin‐2/elafin signals

Trappin‐2 is susceptible to degradation, mostly on the N‐terminal cementoin domain. Elafin is more resistant to proteolytic degradation because of the four disulfide cores. However, it can be degraded by neutrophil elastase, as demonstrated in sputum from cystic fibrosis patients (Guyot et al., 2008; Moreau et al., 2008). In bronchoalveolar lavage fluids from patients with acute lung injury, elafin levels are initially increased, compared with healthy volunteers, but significantly decreased by day 7 after the onset of the injury. This decrease in elafin levels is actually due to 20S proteasome degradation (Kerrin et al., 2013). Variants of elafin that are increasingly resistant to proteolytic degradation have been generated and show increased anti‐inflammatory activity, at least in pulmonary inflammation (Small et al., 2015). House dust mite proteases (Derp1) or bacterial (*P. aeruginosa*) metalloproteinases such as LasB or RgpB proteases, can also proteolytically inactivate elafin (Brown et al., 2003; Guyot et al., 2010).

## 3 PHYSIOLOGICAL ROLE OF TRAPPIN‐2/ELAFIN IN THE GUT

**FIGURE 3:**
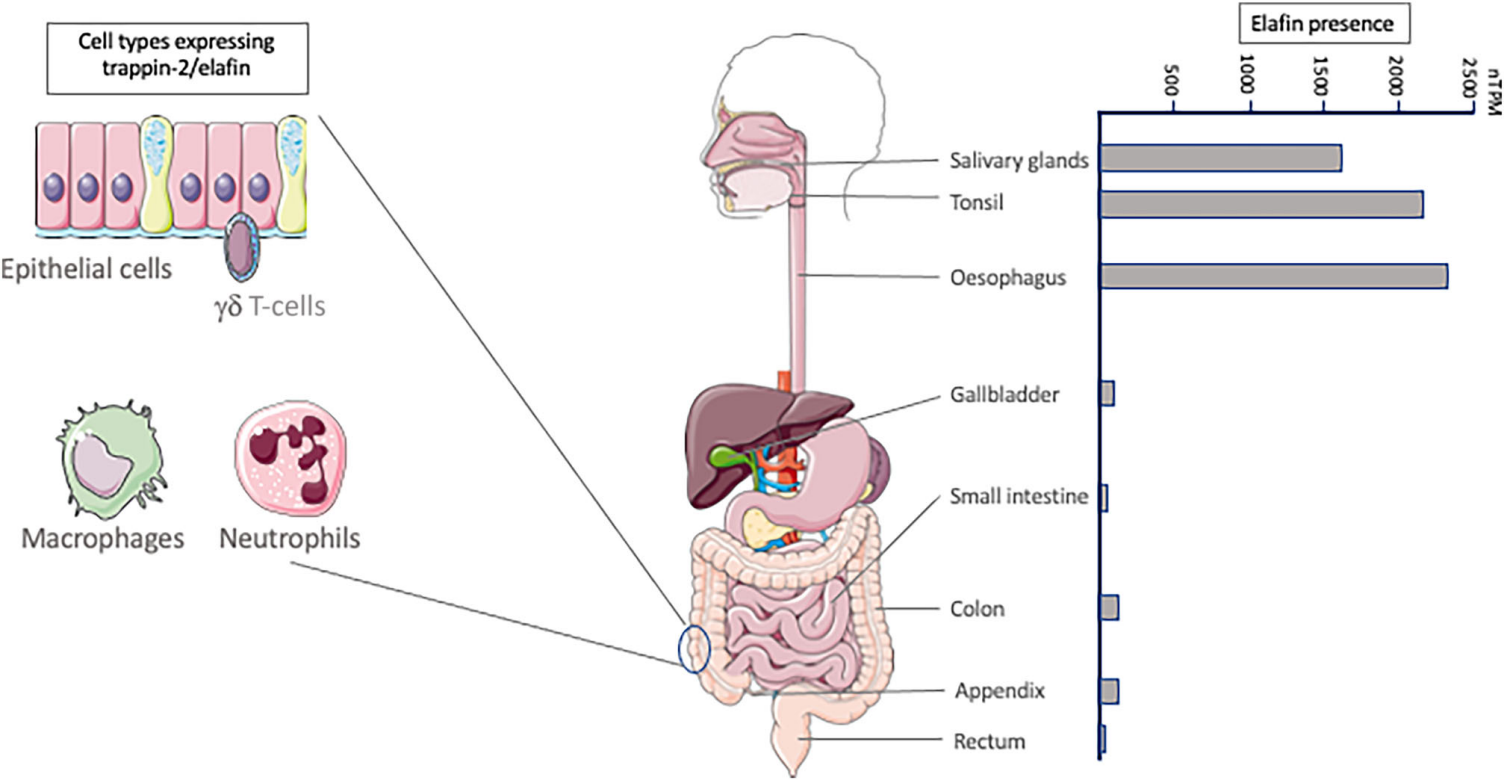
Constitutive expression of elafin along the digestive tract and cellular origin. In colonic tissues, elafin is constitutively expressed by epithelial cells and γδ T‐lymphocytes. During inflammation, infiltrated immune cells such as macrophages and neutrophils also constitute a source of elafin. The schematic representation of mRNA expression level of the *PI3* gene encoding trappin‐2/elafin, in different parts of the gastrointestinal tract in physiological conditions has been extracted from the human protein atlas website (https://www.proteinatlas.org/ENSG00000124102‐PI3/tissue). nTPM, normalized protein‐coding transcripts per million

Elafin and its precursor trappin‐2 are constitutively expressed in human epithelia, including the skin, lung, vagina, oral cavity, and the entire gastrointestinal tract. Because trappin‐2 expression is up‐regulated by trauma, irritation or infection, its functions have been studied mostly in pathological conditions (Sallenave, 2010). However, the constitutive presence of trappin‐2/elafin (see Figure 3) suggests a physiological role at mucosal surfaces. Considering the anchored antimicrobial properties of trappin‐2 and its steady‐state expression in healthy colon mucosa (Motta et al., 2012), it is commonly accepted that trappin‐2/elafin contributes to the physiological mucosal shield against luminal microbes. Indeed, elafin released by intestinal epithelial cells (Galipeau et al., 2014; Motta et al., 2012; Motta, Rolland, et al., 2021) is ideally positioned at the microbe‐epithelial cell interface to play a gate‐keeper role. However, an additional hypothesis on the physiological role of trappin‐2/elafin comes from the work of Caruso et al. (Caruso et al., 2015). In that study, the authors investigated the role of endogenously‐expressed elafin in human mammary epithelial cells. They demonstrated that stable elafin deficiency compromised the ability of epithelial cells to maintain a G0 arrest upon growth factor deprivation. Further, they demonstrated that this effect of elafin was due to its protease inhibitor function (Caruso et al., 2015). This study suggests a role for elafin in physiological control of cell cycle and growth in epithelial cells. However, such physiological role for elafin in gut epithelium has not yet been demonstrated. Long‐term inhibition of physiological trappin‐2/elafin expression could therefore have consequences on the control of proliferative events in intestinal epithelial cells.

## 4 EXPRESSION OF TRAPPIN‐2/ELAFIN IN GUT PATHOLOGIES

The gene coding for trappin‐2/elafin (*PI3*) is highly polymorphic (23 single nucleotide polymorphisms [SNPs] have been identified), mostly in the promoter region (Chowdhury et al., 2006). The fact that most SNPs are in the promoter region suggests that trappin‐2/elafin expression could be very differentially regulated depending on the tissues and also depending on population subgroups that are studied. Indeed, from the mouth to the anus, trappin‐2/elafin expression varies, with a strong presence in the salivary glands, tonsils, and oesophagus and lower expression in the small and large intestine (see Figure 3). In the gut, epithelial cells strongly express trappin‐2/elafin (Motta et al., 2012). In Caco‐2 cells, elafin was up‐regulated by *Lactobacillus plantarum*, through a mechanism involving at least in part, toll‐like receptor (TLR)‐9 activation (Hiramatsu et al., 2019). However, in human primary colonocytes, TLR agonist stimulation did not modify the expression of trappin‐2 mRNA (Motta, Rolland, et al., 2021). Very diverse results have been reported in terms of trappin‐2/elafin expression in healthy compared with pathological tissues in the gut. One important caution though, when considering the expression of a protein in association with a pathological condition, is that such association implies by no means, a role (positive or negative) for such protein, in the pathogenesis of the clinical condition. It could only mean that the regulation of this protein expression is modified by some mediators that are associated with the pathology. This is particularly true for elafin, whose expression seems to be strongly dependent on the phases of the inflammatory response (Sallenave, 2010; Wilkinson et al., 2011). Indeed, in addition to its expression by epithelial cells, trappin‐2/elafin is also expressed by innate immune cells, such as macrophages and neutrophils (Mihaila & Tremblay, 2001; Schmid et al., 2007; Wang et al., 2009) (Figure 3). In terms of the studies describing the expression of trappin‐2/elafin in pathological gut tissues, one has to be cautious in claiming the involvement of trappin‐2/elafin in such pathologies.

### 4.1 Trappin‐2/elafin expression in IBD

Microarray studies of human colonic biopsies have determined that trappin‐2 transcripts were up‐regulated in tissues from ulcerative colitis patients (Eriksson, Flach, et al., 2008; Flach, et al., 2006). The authors have observed that the up‐regulation of trappin‐2 seems to follow the progression of the disease. Indeed, trappin‐2 transcript was elevated in the proximal part of the colon in patients with total colitis, but not in patients with left‐side colitis. One could hypothesize that recently inflamed and damaged tissues are trying to respond to injury by up‐regulating the expression of trappin‐2/elafin, a dual protease inhibitor and antimicrobial agent. However, tissues that are already in a state of chronic inflammation (distal rectal tissues in ulcerative colitis patients) might not be any longer capable of mounting such a protective response. Such hypothesis is supported by the findings of Zhang et al. who have investigated the clinical significance of trappin‐2/elafin expression (Zhang et al., 2017). They observed that in active ulcerative colitis, colonic trappin‐2 mRNA decreased significantly, but in the contrary increased upon remission. This study clearly established that trappin‐2 expression was significantly lower in inflamed colonic mucosa of ulcerative colitis patients compared with healthy controls, or compared with non‐inflamed colonic mucosa of ulcerative colitis patients (Zhang et al., 2017). It actually confirmed a previous report describing a decreased trappin‐2 mRNA expression in tissues from ulcerative colitis patients compared with healthy controls. Interestingly, in that study, trappin‐2 mRNA was followed by in situ hybridization and revealed that in healthy controls, trappin‐2 transcripts are mostly synthesized by intestinal epithelial cells (Motta et al., 2012). In ulcerative colitis patients, elafin protein expression was reported to originate both from the mucosa and the lamina propria in infiltrated inflammatory cells (Schmid et al., 2007; Wang et al., 2020). Colon crypt analysis and tissue in situ hybridization reported that trappin‐2/elafin mRNA expression was reduced in intestinal epithelial cells from IBD patients (Motta et al., 2012; Motta, Rolland, et al., 2021).

Similar microarray studies have been performed in Crohn's disease patients, demonstrating that trappin‐2 expression was increased in non‐inflamed rectal mucosa but decreased in inflamed colonic mucosa. However, no correlation between disease activity and trappin‐2 transcript expression was revealed in that study (Eriksson, Jennische, et al., 2008). Similarly, the expression of trappin‐2 in the inflamed colonic mucosa of Crohn's disease patients was found to be lower than that in non‐inflamed mucosa from Crohn's disease patients or from healthy controls (Motta et al., 2012; Zhang et al., 2017). Interestingly, another recent study has demonstrated a negative correlation between trappin‐2 mRNA expression in colonic tissues from Crohn's disease patients and the presence of strictures or the expression of fibrogenic factors (Wang et al., 2020). Taken together, these studies report a defect in colonic elafin expression associated with Crohn's disease.

For all the above studies, trappin‐2/elafin mRNA expression has been studied in colonic tissues. In the search for biomarkers, circulating elafin in sera has also been measured in IBD patients and appears to be a relevant biomarker for some IBD features. One recent study has identified an association between high circulating elafin levels in serum from Crohn's disease patients and the presence of intestinal strictures. Interestingly, the authors observed that intestinal stricture was associated at the same time with increasing sera levels of elafin and reduced intestinal expression of elafin (Wang et al., 2020). This suggests that depending on the source of elafin (possibly circulating cells relative to intestinal epithelium), the levels of elafin reflect different disease association. One can also infer that elafin is not involved in the pathogenesis of stricture, but rather could be used as a serum biomarker for this pathological event.

Taken together, both in Crohn's disease and in ulcerative colitis, local expression of trappin‐2/elafin seems to be associated with a healthier mucosa. Although a relationship with overall clinical disease activity was not found in all studies, a consistent negative correlation between trappin‐2/elafin and tissue damage was observed (Wang et al., 2020; Zhang et al., 2017). A local decrease in trappin‐2 expression was associated with chronically damaged mucosa, suggesting that exogenous delivery of trappin‐2/elafin would have beneficial input. Interestingly, a recent study suggested that the protective effects of *Lactobacillus plantarum* in injured intestinal epithelial cells could be mediated by an increased expression and secretion of elafin by such cells. This regulation of trappin‐2/elafin expression by a probiotic in intestinal epithelial cells was under the control of TLR‐9 (Hiramatsu et al., 2019). This nicely complicates the picture of trappin‐2/elafin regulation, suggesting that it can be influenced by food‐grade bacteria.

### 4.2 Trappin‐2/elafin expression in celiac disease

Considering the differential expression of elafin in IBD described above, the question about trappin‐2/elafin expression in other pathologies accompanied by intestinal inflammation, such as celiac disease, seems relevant. Only one study so far has investigated elafin protein expression in the small intestine of patients with celiac disease (Galipeau et al., 2014). This study reported that elafin protein expression was weaker in the intestinal mucosa of patients with active celiac disease, compared with non‐celiac controls. Importantly, elafin expression in the intestine of celiac disease patients in remission (1‐year gluten‐free diet) was not significantly different from non‐celiac, suggesting here again that decreased elafin expression is associated with damaged mucosa.

### 4.3 Trappin‐2/elafin expression in colorectal cancer

Differential expression of elafin has been reported in tumours depending on the origin of tissues that were sampled. It is overexpressed in oesophageal cancer and certain breast and ovarian cancers (Labidi‐Galy et al., 2015; Yamamoto et al., 1997), but it is down‐regulated in other breast or ovarian tumours (Caruso et al., 2014). Only one study so far has looked at elafin expression in colorectal cancer, demonstrating a higher expression of trappin‐2 mRNA (twofold) in colorectal cancer samples, compared with adjacent non‐cancerous tissues. Interestingly, elafin expression was increased in tissues at early stages of colorectal cancer development. As discussed by the authors, this increased elafin expression could be due to inflammatory cell infiltration. No correlation was found between elafin expression and tumour invasive stage or metastasis (Liu et al., 2019). No experimental results support pro‐ or anti‐oncogenic properties for elafin in intestinal epithelial cells, thus far.

## 5 TRAPPIN‐2/ELAFIN PROTECTS AGAINST INTESTINAL INFLAMMATION

Most of the research that has been done on trappin‐2/elafin relates to mucosal defence and mucosal functions, a large attention having been given to the lung mucosa. However, a growing body of evidence also suggests a role for trappin‐2/elafin in the intestinal mucosa. Considering the pleiotropic effects of trappin‐2/elafin on mechanisms related to inflammation (see Figure 2), converging evidence pointed to a role for this protein in inflammatory bowel diseases such as Crohn's disease or ulcerative colitis and potentially in celiac disease. Such role is supported by key studies that have demonstrated a protective effect for trappin‐2/elafin delivery in different models of acute or chronic intestinal inflammation.

### 5.1 Acute inflammation

Several studies have demonstrated a protective role for trappin‐2/elafin in acute models of inflammation. Mice do not express trappin‐2/elafin. Therefore, in order to demonstrate the effects of trappin‐2/elafin in mouse models of gut acute inflammation, early studies have used transgenic mice that express trappin‐2/elafin in different tissues, including the gut mucosa (Motta et al., 2011; Sallenave et al., 2003; Zaidi et al., 1999). Acute colitis was induced in these mice by the addition of dextran sodium sulfate (3%) to the drinking water for 7 days, or by the intracolonic administration of trinitrobenzene sulfonic acid diluted in 50% ethanol. For both models, 7 days after the induction of colitis, elafin‐expressing mice were protected from colitis, showing a decreased infiltration of inflammatory cells, decreased tissue damage, inhibition of pro‐inflammatory cytokines such as IL‐6 and IL‐17 or chemokines such as KC (the mouse homlogue of CXCL1), CCL3 (MIP‐1α), CXCL2 (MIP‐2), and CCL5 (RANTES) (Motta et al., 2011). Elafin expression in transgenic mice also inhibited NFκB activation associated with dextran sodium sulfate‐induced colitis. The use of transgenic mice that express elafin ubiquitously could not differentiate between the systemic effects of trappin‐2/elafin and the local (gut) expression. The authors have then tested the effects of intracolonic adenoviral delivery of elafin in the same model of acute colitis, demonstrating that colonic adenovirus‐elafin expression in wild‐type mice reproduces the elafin protection observed in transgenic mice (Motta et al., 2011). Other studies have demonstrated the protective role of trappin‐2/elafin delivery in the acutely inflamed gut, using food‐grade bacteria as carriers, being genetically modified to express and secrete human recombinant trappin‐2 (Bermúdez‐Humarán et al., 2015; LeBlanc et al., 2013; Motta et al., 2012). Lactic acid bacteria (*Lactococcus lactis* or *Lactobacillus casei*) were recombined for the expression of *human* trappin‐2 and were given orally to mice in which acute colitis was induced. Human elafin protein was detected in the colonic mucosa of mice that were orally treated with such recombinant bacteria. Both *L. lactis and L. casei* recombinant for trappin‐2 conferred a strong protection against the acute colitis induced by dextran sodium sulfate (Motta et al., 2012). This effect was more pronounced and wider in terms of the inflammatory parameters that were followed than the anti‐inflammatory effects induced by lactic acid bacteria recombinant for IL‐10 or TGF‐β (Bermúdez‐Humarán et al., 2015). Strikingly, genetic modification of the trappin‐2‐recombinant *L. lactis* aiming at improving trappin‐2/elafin production further enhanced the protection against acute colitis (Bermúdez‐Humarán et al., 2015). This strongly supports the causal link between the amount of trappin‐2/elafin present in the gut and the achieved protection. Recently, an abstract from the 14th Congress of the European Crohn's and Colitis Organization also reported that the probiotic strain *E. coli* Nissle 1917 recombined with elafin, protected mice from dextran sodium‐sulfate‐induced acute colitis (Teng et al., 2019). In addition to animal models of inflammation, acute exposure of human intestinal epithelial cells to elafin in vitro demonstrated protection against TNFα‐induced increased permeability. Exogenous addition of elafin to human intestinal epithelial cells also reduced TNFα‐induced increase in CXCL8 secretion. These results provided evidence that “inflamed” human intestinal epithelium is also protected by the exogenous addition of elafin (Motta et al., 2011).

### 5.2 Chronic inflammation

A chronic model of inflammation mimicking what is observed in IBD patients experiencing periods of remission and relapses was devised, exposing mice to successive cycles of dextran sodium sulfate. In this model, daily oral treatments with trappin‐2‐expressing *L. lactis* protected from inflammation. Mice showed decreased tissue damage and inflammatory cell infiltration, together with an increased mucosa healing rate (Motta et al., 2012). In another model of chronic intestinal inflammation mediated by adaptive transfer of CD45RB^High^ subpopulation of CD4^+^ cells (Ostanin et al., 2009), oral treatment of mice with trappin‐2‐recombinant *L. lactis* strongly protected the mice from mucosal damage (Motta et al., 2012). This model of colitis is entirely T‐cell dependent and allows the investigation of the effects of therapeutic approaches on the immune component of mucosal inflammation. The protective effect of trappin‐2/elafin delivery in this chronic model of inflammation demonstrated the effects that trappin‐2/elafin could exert on specific immunological mechanisms. To evaluate the effects of trappin‐2‐expressing *L. lactis* in an IBD environment, human intestinal epithelial cells have been stimulated with biopsy supernatants from IBD patients. This stimulation increased epithelial permeability and the expression of the CXCL8 and CCL2 (MCP‐1) chemokines, which were all reduced by co‐incubation with trappin‐2‐expressing *L. lactis*, but not with wild‐type *L. lactis* (Motta et al., 2012). Importantly, this result provides direct evidence of the protective effects of trappin‐2/elafin delivery against epithelial dysfunctions caused by mediators associated with IBD patients.

### 5.3 Gluten sensitization

Trappin‐2/elafin delivery has also proven its efficacy against intestinal inflammation induced by gluten sensitization. In gliadin‐sensitized NOD/DQ8 mice, a model of gluten sensitivity, oral treatment with trappin‐2‐recombinant *L. lactis* protected from intraepithelial lymphocytosis and from intestinal barrier dysfunctions (Galipeau et al., 2014). In the same study, the authors also demonstrated that trappin‐2 slowed down the kinetics of appearance of gliadin‐derived immunological peptide.

Taken together, these data demonstrated that trappin‐2/elafin delivery at the surface of an inflamed intestinal mucosa exerted a protective role, in a wide variety of models, with diverse aetiology. This suggests that trappin‐2/elafin is able to interfere with several inflammatory pathways, having diverse potential targets involved in the complex mechanisms of intestinal inflammation. The details of such targets in the gut are set out in the paragraph below.

## 6 TARGETS OF TRAPPIN‐2/ELAFIN IN INTESTINAL INFLAMMATION

### 6.1 Proteases

Elafin and its precursor trappin‐2 have been thought, initially, to have the most restricted spectrum of anti‐protease activity, being merely described as neutrophil elastase and proteinase‐3 inhibitors. Complementary studies have nonetheless demonstrated that trappin‐2/elafin can exert inhibitory effects on other proteases that are present in the inflamed gut, including microbial proteases. Diverse roles have been demonstrated for trappin‐2/elafin‐sensitive proteases in gut inflammation and are reviewed below.

#### 6.1.1 Neutrophil elastase and proteinase‐3

Neutrophil elastase and proteinase‐3 are two proteases present in azurophil granules of neutrophils and that are released upon neutrophil activation. They are closely related, first because of their cellular origin, second because they have affinities for similar substrates, and third because both proteases can be inhibited by trappin‐2/elafin in the same concentration range (Table 1). Obviously, upon inflammatory cell (neutrophil) infiltration in inflamed tissues (as observed in IBD, celiac disease, or intestinal infection), both neutrophil elastase and proteinase‐3 are up‐regulated and both could constitute targets for trappin‐2/elafin.

In the gut, neutrophil elastase and proteinase‐3 exert different effects and through a variety of mechanisms, all including the proteolytic processing of other proteins. They both can cleave and activate proteinase‐activated receptors (PARs)‐1 and ‐2 (Mihara et al., 2013; Muller et al., 2007; Ramachandran et al., 2011), which are known to affect a number of intestinal physiological functions, both PAR1 and PAR2 being clearly pro‐inflammatory and pro‐nociceptive (Buresi et al., 2001; Cenac et al., 2002, 2004, 2005; Desormeaux et al., 2018; Hyun et al., 2008; Motta, Rolland, et al., 2021; Vergnolle, 2004; Vergnolle et al., 2010). Both neutrophil elastase and proteinase‐3 can enhance the chemotactic effect of CXCL8 upon its cleavage (Padrines et al., 1994), and proteinase‐3 also activates IL‐1, IL‐18, and TNFα (Coeshott et al., 1999; Robache‐Gallea et al., 1995; Sugawara et al., 2001). Neutrophil elastase is known to affect barrier function both by activating PARs and by cleaving E‐cadherin (Chin et al., 2003, 2008; Nava et al., 2013). Overly active neutrophil elastase has also been shown to have a deleterious effect on extracellular matrix remodelling. Indeed, in intestinal tissues of ulcerative colitis patients, neutrophil elastase seemed to contribute to an impaired mucosal repair (Kuno et al., 2002). Recently, it was reported that human neutrophil elastase was able to cleave biological drugs used in IBD patients (infliximab, adalimumab, etanercept, and vedolizumab), impairing the TNFα‐neutralizing capacity of such therapeutic agents (Curciarello et al., 2020). This study demonstrated that elastase can affect the integrity or therapeutic monoclonal antibodies as well as therapeutic fusion proteins such as etanercept, suggesting that the high content of neutrophil elastase in tissues from ulcerative colitis patients could contribute to an impaired response to biological drugs.

However, proteinase‐3 and neutrophil elastase are also known for their role in controlling inflammation and infectious processes. Neutrophil elastase contained in neutrophil extracellular traps, contributes to the capture and neutralization of pathogenic microorganisms (Brinkmann et al., 2010). Both elastase and proteinase‐3 can inactivate pro‐inflammatory cytokines such as IL‐6 by proteolytic cleavage (see Vergnolle, 2016). Proteinase‐3 is pro‐apoptotic through the activation of pro‐caspase‐3 in neutrophils or endothelial cells (Pendergraft et al., 2004), thereby potentially terminating inflammatory signals in neutrophils. Although E‐cadherin is cleaved by neutrophil elastase resulting in an increased intestinal permeability (Ginzberg et al., 2001), the E‐cadherin peptide resulting from this cleavage is also capable of stimulating wound healing (Gordon et al., 2019). The hyperactivity of neutrophil elastase and proteinase‐3 that is associated with IBD may well be a double‐edged sword, which participates concomitantly, and through different mechanisms of action, in the inflammatory overflow and to its control.

#### 6.1.2 Epithelial elastase

Studies that have used in situ zymography techniques to localize elastolytic activity in tissues from IBD patients have reported that elastolytic activity was not only observed in the lamina propria but also strongly associated with the epithelium (Motta et al., 2012; Motta, Rolland, et al., 2021). These studies have prompted researchers to investigate the possible presence of active elastases in intestinal epithelium. An epithelial form of elastase was discovered: Ela2A, which can be synthesized and secreted by human intestinal epithelial cells. Ela2A released from intestinal epithelium was inhibited by elafin and was up‐regulated in tissues from IBD patients. In vivo up‐regulation of epithelial Ela2A caused colonic mucosa inflammation, characterized by increased permeability, tissue damage, production of pro‐inflammatory cytokines and chemokines, and inhibition of gene expression of mediators involved in epithelial protection and repair. Epithelial Ela2A hyperactivity was sufficient to lead to a leaky epithelial barrier, which was inhibited by the addition of exogenous elafin (Motta, Rolland, et al., 2021). This recent result adds to the list of elafin intestinal targets, an epithelial elastase, which seems heavily involved in mucosal inflammation, particularly in the context of IBD.

#### 6.1.3 Microbial proteases

A recent study that has looked at a reference set of bacterial serine protease sequences available at the National Center for Biotechnology Information has identified 285 putative serine protease sequences from the human gut microbiota, assigned to 56 different genera, from five phyla (Kriaa et al., 2020). It is likely that, among those serine proteases, some might be inhibited by trappin‐2/elafin. Indeed, elastolytic activity and proteinase‐3 up‐regulation were reported in faecal samples from IBD or celiac disease patients (Caminero et al., 2016; Jablaoui et al., 2020). Increased elastase activity was further defined as a marker in patients preceding the onset of ulcerative colitis (Galipeau et al., 2020). Whether such elastolytic activity comes from the host (potentially epithelial Ela2A) or the microbiota is not yet clear. However, it is certain that microbial communities are able to produce elastolytic activity. *Pseudomonas aeruginosa* is an opportunistic pathogen commonly found in human microbiota samples and can be harvested from celiac disease or IBD patients (Caminero & Verdu, 2019). *P. aeruginosa* produces a form of elastase (pseudolysin or LasB), whose structure is very close to that of human neutrophil elastase. *P. aeruginosa* elastase could participate in mucosal inflammation. Indeed, in the context of celiac disease, *P. aeruginosa* elastase synergizes with gluten to induce more severe inflammation (Caminero et al., 2019). Also, *P. aeruginosa* arginyl peptidase is a target for trappin‐2/elafin, demonstrating that both trappin‐2 and elafin contribute to antibacterial activity against *P. aeruginosa* and its secreted peptidase (Bellemare et al., 2008, 2010). In vitro, addition of exogenous trappin‐2/elafin exogenous addition reduced the formation of *P. aeruginosa* biofilms (Bellemare et al., 2010). Therefore, it is likely that trappin‐2/elafin released by intestinal epithelial cells could control gut mucosal biofilms, as it has been demonstrated for other epithelial factors involved in mucosal proteolytic homeostasis (Motta et al., 2019; Motta, Wallace, et al., 2021).

### 6.2 Microbes

No studies have yet investigated whether trappin‐2/elafin would target intestinal microbiota. However, considering the extended antimicrobial properties that have been described for trappin‐2/elafin, including against pathogenic bacteria such as pathogenic *E. coli* or *P. aeruginosa*, that are known to be present in the inflamed gut (see above), it is logical to think that variations in mucosal expression and secretion of trappin‐2/elafin could alter the composition of microbiota. In a model of colitis induced by dextran sodium sulfate, mice transgenic for the expression of trappin‐2 had lower proteobacteria and higher firmicute abundance (Vergnolle et al. personal unpublished observations), compared with wild‐type littermates, suggesting that colitogenic bacterial communities would be suppressed by the presence of trappin‐2/elafin. Further studies are necessary to fully understand the effects of intestinal expression of trappin‐2/elafin could have on mucosa‐attached biofilms, in health and disease. However, it is clear from studies that have investigated the antimicrobial properties of trappin‐2/elafin that they could exert antimicrobial properties against a variety of viruses, bacteria, parasites, or fungi that may be present in the gut and that could enter host tissues.

### 6.3 NFκB/AP1 signalling

The inhibitory role that trappin‐2/elafin has been shown to exert on NFκB or AP1 present in monocytes and macrophages can obviously occur also in an inflamed intestinal mucosa, where such inflammatory cell types are known to be present. Indeed, in vivo studies have demonstrated that colitis‐induced NFκB activity was modulated in the intestine of transgenic mice expressing elafin, compared to wild‐type mice. This inhibition was accompanied by a decrease in all inflammatory parameters, suggesting that the inhibitory effects of elafin on NFκB pathways, can prevent the development of inflammation. It is unclear whether this NFκB ‐targeted elafin effect is due to an inhibition of NFκB in inflammatory cells or in resident cells such as intestinal epithelium. Experimental data suggest that activation of NFκB in intestinal epithelial cells could also be a target for trappin‐2/elafin (Motta et al., 2011).

### 6.4 Transglutaminases

Transglutaminases are members of a family of enzymes that catalyse the covalent cross‐linking between proteins, by forming isopeptide bonds. In gastroenterology, they are particularly relevant as they are involved in celiac disease. One enzyme in particular, the tissue transglutaminase has attracted most attention for its role in gluten deamidation and as a target autoantigen. However, transglutaminases are also actively involved in intestinal mucosa healing processes (D'Argenio et al., 2005; Siegmund & Zeitz, 2005). Abnormal patterns of transglutaminases have been shown to contribute to the course of ulcerative colitis or Crohn's disease. By being expressed in epidermal epithelial cells, for example, keratinocyte transglutaminase is involved in skin barrier integrity by cross‐linking structural proteins (D'Argenio et al., 1995; Siegmund & Zeitz, 2005). Upon tissue reconstruction, they stabilize a fibrous extracellular matrix that sets the stage for re‐epithelialization (Telci & Griffin, 2006). There is, thus, good evidence that transglutaminases are key positive elements of intestinal mucosal reconstruction and integrity. Being a substrate for transglutaminases (see above), trappin‐2/elafin may contribute to the effects of transglutaminases in intestinal inflammatory situations. Although not demonstrated yet, it is logical to hypothesize that in an inflamed intestinal mucosa, by being anchored to the extracellular matrix in a transglutaminase‐dependent fashion, trappin‐2 may protect this nascent matrix support from proteolysis and from microbial invasion. This, of course, would have to be fully demonstrated by specific studies looking at the effects of trappin‐2 delivery in intestinal mucosa, with or without expression of transglutaminases.

In the context of celiac disease, elafin was able to significantly slow down the kinetics of deamination of the 33‐mer gliadin peptide into a more immunogenic form by tissue transglutaminase. Elafin was not as efficient as tridegin, a known transglutaminase inhibitor (Galipeau et al., 2014). Thus, it was suggested that in celiac disease, as a transglutaminase substrate, elafin could decrease (even slightly) the rate of tissue transglutaminase‐induced production of immunogenic peptides, by substrate competition.

## 7 WHAT IS THE THERAPEUTIC POTENTIAL FOR TRAPPIN‐2/ELAFIN DELIVERY IN THE INFLAMED GUT?

Preclinical studies have already demonstrated the efficacy of trappin‐2/elafin delivery in the gut in protecting against both acute and chronic mucosal inflammation (see above). This leads directly to the question of its therapeutic potential in human intestinal diseases.

### 7.1 Trappin‐2/elafin: A safe molecule

The first question that must be answered in the search for arguments in favour or against the use of trappin‐2/elafin in humans is its safety. As previously described, trappin‐2/elafin has a restricted spectrum of protease inhibition (Moreau et al., 2008; Sallenave, 2010). For instance, it does not inhibit proteases of the coagulation cascade (Table 1). Despite the ability of trappin‐2/elafin to inhibit some pancreatic enzymes in vitro (Ela2A, for instance), elafin delivery to mice did not cause any signs of pancreatic enzyme insufficiency or weight loss. Intravenous, intranasal, or aerosol administration of elafin to a number of different species has been shown to be safe and, actually, protective against a number of cardiovascular or lung clinical conditions (Shaw & Wiedow, 2011). Phase I and Phase II clinical trials in which patients have received intravenous elafin have been performed and reported favourable safety profiles for elafin, with no drug‐related adverse events (Phase II trials have not been performed for a gut indication, but for coronary artery bypass surgery indications). Therefore, elafin delivery in the gut appears to have quite a safe profile. Even in terms of possible carcinogenesis, despite the reported slight up‐regulation (only twofold) of elafin expression in colorectal cancer samples (Liu et al., 2019), tumour‐suppressive properties have been suggested for elafin in other forms of carcinoma (mammary), where it has been shown to counteract the mitogenic effects of up‐regulated neutrophil elastase (Caruso et al., 2015).

### 7.2 Pleiotropic effects of trappin‐2/elafin on inflammation‐associated mucosal dysfunctions

**FIGURE 4:**
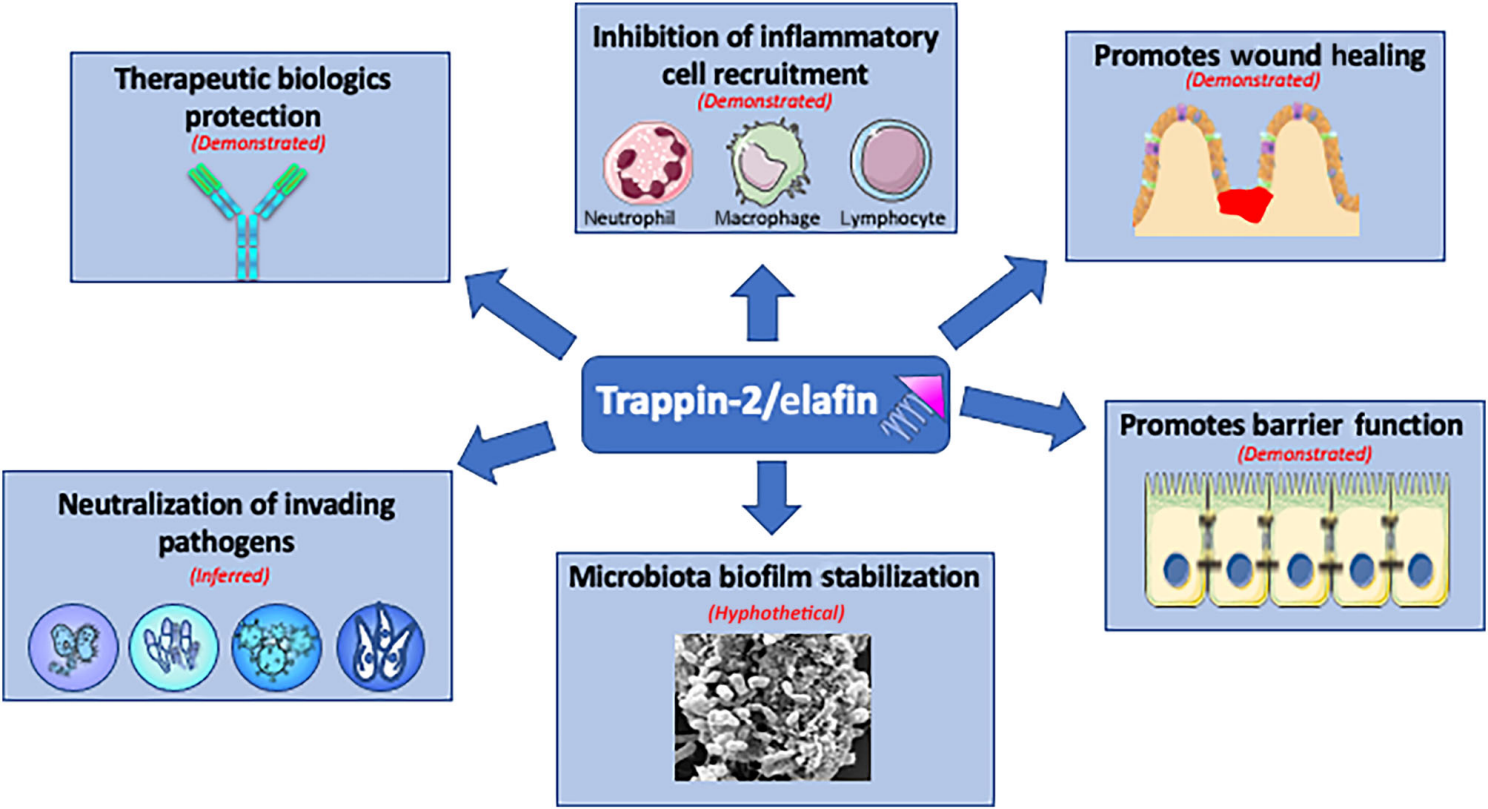
Potential effects of trappin‐2/elafin delivery on mucosal dysfunction, associated with intestinal inflammation. Experimentally demonstrated, inferred or hypothetical roles are shown.

The second question to ask when assessing the therapeutic potential of an endogenously expressed protein, is its efficacy. Trappin‐2/elafin appears to have the potential of acting at several levels, within several pathways implicated in intestinal inflammation and associated mucosal dysfunctions. The pleiotropic nature of its effects (protease inhibition, nuclear factor control, antimicrobial properties, and as a transglutaminase substrate) confers to trappin‐2/elafin a Swiss army knife role, acting on several major features of chronic inflammatory disorders in the intestine (see Figure 4).

#### 7.2.1 Inhibiting inflammatory cell recruitment

Through several of the different pathways described above, trappin‐2/elafin delivery is able to inhibit granulocyte recruitment to the site of inflammation, as shown in different murine models of colitis (Motta et al., 2012). As indicated above in Section 2.4, trappin‐2/elafin inhibits NF‐kB and AP‐1‐dependent activation of inflammation, which both control a number of mediators involved in the chemoattraction of inflammatory cells (Mussbacher et al., 2019). Motta et al. have shown that TNFα‐stimulated intestinal epithelial cells produce lower amounts of the chemokines CXCL‐8, CCL2 and CXCL10, when treated with elafin, thereby reducing their potential to attract immune cells (Motta et al., 2012). Furthermore, elafin inhibits target proteases (see Table 1) that are able to cleave some protease‐activated receptors (PARs). PARs are GPCRs playing major roles in inflammation. Some of these cleavages result in an increase in endothelial cell barrier permeability. This is the case for proteinase 3‐mediated cleavage of PAR1 that activated the MAPK signalling pathway and increased permeability in endothelial cells (Mihara et al., 2013). Therefore, these results suggest that trappin‐2/elafin may inhibit leukocyte extravasation by inhibiting proteinase 3‐dependent cleavage of endothelial PAR1. In addition to PAR1, PAR2 has important pro‐inflammatory functions during colitis (Hyun et al., 2008), particularly in the recruitment of inflammatory cells (Hyun et al., 2010). Neutrophil elastase has been shown to activate PAR2 in a biased manner (Ramachandran et al., 2011). As PAR2 is required to promote rolling and adhesion of leukocytes, including in mouse models of colitis (Hyun et al., 2008; Vergnolle, 1999), trappin‐2/elafin may decrease this process by inhibiting the cleavage and downstream activation of PAR2 by neutrophil elastase.

#### 7.2.2 Consolidating intestinal barrier function

Altered barrier function is a major feature of chronic inflammation, constantly feeding a mucosal inflammatory response. Protease signals are major components of intestinal barrier degradation, acting by direct effects on junction proteins, PAR signalling or mucus degradation (Vergnolle, 2016). Trappin‐2/elafin control of deleterious proteolytic activity is therefore a potent signal to consolidate barrier function upon mucosal inflammation. Indeed, trappin‐2/elafin inhibits the epithelial elastase Ela2A, which has been shown to be hyper‐active in IBD. Ela2A is responsible for the degradation of proteins involved in cellular junctions leading to leaky epithelial barriers (Motta, Rolland, et al., 2021). The involvement of both PAR1 and PAR2 activation in increased intestinal permeability has been demonstrated (Cenac et al., 2002, 2004; Chin et al., 2003, 2008). Here again, by inhibiting PAR‐activating proteases such as elastases and proteinase‐3, trappin‐2/elafin could consolidate epithelial barrier function. Importantly, trappin‐2/elafin delivery has been shown to strengthen barrier function both in vitro and in vivo (Motta et al., 2011).

#### 7.2.3 Promoting wound healing

Anchoring of trappin‐2 by transglutaminase on fibronectin or other extracellular matrix components that are part of matrix assembly, in wound repair may favour tissue healing through different mechanisms. First, being anchored and active as a protease inhibitor, trappin‐2/elafin could inhibit neutrophil elastase, proteinase‐3, Ela2A, or microbial proteases, which otherwise might degrade newly formed extracellular matrix. Through that mechanism, trappin‐2/elafin could counteract a protease‐dependent delayed tissue repair. Anchored trappin‐2 in nascent repaired matrix may also exert antimicrobial properties, preventing further microbe entry and consequent activation of the innate immunity system.

#### 7.2.4 Neutralizing invading pathogens

Either in damaged mucosa or in nascent tissue repair zones, trappin‐2/elafin can exert its antimicrobial properties. Chronic inflammation is associated with microbial translocation and tissue invasion with pathogens. Locally released trappin‐2/elafin could importantly contribute to the control and neutralization of invading pathogens, either by direct antimicrobial effects or by regulating adaptive immunity (Roghanian, Drost, et al., 2006; Roghanian, Williams, et al., 2006) (see also Section 2).

#### 7.2.5 Microbiota stabilization

So far, no published study has directly established an effect for trappin‐2/elafin on the composition and phenotypic behaviour of intestinal microbiota. However, the role of epithelial proteases on the physical organization, metabolism and pathogenicity of intestinal microbiota has clearly emerged and is particularly exemplified by thrombin activity released by intestinal epithelium (Motta et al., 2019; Motta, Wallace, et al., 2021). As it is able to inhibit epithelial proteases such as elastase‐2 (Motta, Rolland, et al., 2021), trappin‐2/elafin would affect intestinal biofilms, although such effects could be detrimental or protective. However, considering its antimicrobial effects against potential gut pathogens, it could be hypothesized that trappin‐2/elafin delivery could modify biofilm composition and prevalence of pathogens. Although plausible, a role for trappin‐2/elafin in microbiota stabilization would have to be fully investigated.

#### 7.2.6 Inhibition of the degradation of therapeutic antibodies

Recent studies have reported that high levels of mucosal neutrophil elastase or epithelial elastase Ela2A can degrade therapeutic antibodies leading to a loss of function of such biological agents (Curciarello et al., 2020) (Deraison et al., personal communication). In particular, this was observed for biological agents that are used in the treatmentof IBD, such as infliximab, adalimumab, etanercept and vedolizumab. Because it can inhibit both neutrophil elastase and Ela2A, trappin‐2/elafin delivery could be envisioned, in addition to all their effects on inflammation‐associated dysfunctions, as a companion treatment that would foster the effects of currently used biological agents, by preventing their degradation.

## 8 CONCLUSION

A large body of evidence points to trappin‐2/elafin as exerting important effects in gastrointestinal pathologies and particularly, in inflammation‐associated diseases. This does not mean that trappin‐2/elafin is a factor involved in the pathogenesis of intestinal inflammation. Indeed, no trappin‐2/elafin mutant(s) has been associated with IBD or celiac disease. However, from all the studies that have been reported so far, it appears that trappin‐2/elafin plays its role in trying to control, locally, the inflammatory response. It could be that in chronic inflammation or severe tissue damage, such as that in IBD or in un‐managed celiac disease, these local effects of trappin‐2/elafin are overwhelmed. Mucosal delivery of trappin‐2/elafin could therefore be envisioned for such patients, as a complementary approach to current treatments, fostering tissue repair and mucosal return to homeostasis. In this context, gut inflammatory pathologies in the gut might be easier to treat than those in other mucosal surfaces, as several delivery systems, including genetically engineered probiotics or commensals, are readily available to carry such small proteins through to the targeted mucosa in future clinical studies. However, these live biotherapeutic agents must be safe for the environment, unable to disseminate outside the human body and unable to transfer its exogenous gene to the environmental bacteria (Barra et al., 2020). Bio‐containing genetically modified bacteria is a very promising approach to new therapies for patients suffering from intestinal diseases and has already been used in clinical studies (Braat et al., 2006; Steidler et al., 2009).

### 8.1 Nomenclature of targets and ligands

Key protein targets and ligands in this article are hyperlinked to corresponding entries in http://www.guidetopharmacology.org, and are permanently archived in the Concise Guide to PHARMACOLOGY 2021/22 (Alexander, Christopoulos et al., 2021; Alexander, Fabbro et al., 2021a, b; Alexander, Kelly et al., 2021).

## AUTHOR CONTRIBUTIONS

NV, KR, and PL conceived the review, and NV, CD, and CB collected bibliography, designed figures and tables, and wrote the manuscript. CD, CB, PL, and NV revised the manuscript.

## CONFLICT OF INTEREST

KR is an employee of Nexbiome Therapeutics. NV and PL are inventors on a patent (9688742). All other authors have no conflict of interest.

## Data Availability

Data sharing is not applicable as no new data were generated.
